# Jack of all trades, master of each: the diversity of fibroblast growth factor signalling in eye development

**DOI:** 10.1098/rsob.210265

**Published:** 2022-01-12

**Authors:** Neoklis Makrides, Qian Wang, Chenqi Tao, Samuel Schwartz, Xin Zhang

**Affiliations:** Departments of Ophthalmology and Pathology and Cell Biology, Columbia University, New York, NY, USA

**Keywords:** FGF, optic cup, lacrimal gland, lens, retina, ciliary margin

## Abstract

A central question in development biology is how a limited set of signalling pathways can instruct unlimited diversity of multicellular organisms. In this review, we use three ocular tissues as models of increasing complexity to present the astounding versatility of fibroblast growth factor (FGF) signalling. In the lacrimal gland, we highlight the specificity of FGF signalling in a one-dimensional model of budding morphogenesis. In the lens, we showcase the dynamics of FGF signalling in altering functional outcomes in a two-dimensional space. In the retina, we present the prolific utilization of FGF signalling from three-dimensional development to homeostasis. These examples not only shed light on the cellular basis for the perfection and complexity of ocular development, but also serve as paradigms for the diversity of FGF signalling.

## Graphical abstract

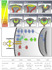

## 1. Introduction

From so simple a beginning endless forms most beautiful and most wonderful have been, and are being, evolved. Charles Darwin, *The Origin of Species*

The endless diversity of living organisms inspired Charles Darwin to discover the theory of evolution. However, how this complex array of diversity emerges at the cellular level eluded his theory. This question has since fascinated developmental biologists for the last two centuries, culminating in the identification of many genes and proteins necessary for development. Remarkably, just as Darwin observed that endless forms evolve from a shared, simple beginning, numerous studies have demonstrated that these molecular players are orchestrated by only a handful of major signalling pathways. Thus, it is crucial to understand how such a limited vocabulary is used to construct the language of life.

Fibroblast growth factor (FGF) signalling is one of the foundational pathways that influences almost every aspect of the biological system [1]. First discovered for its mitogenic potential to promote proliferation of fibroblast cells, FGF signalling is now appreciated not only for its role in embryonic development, but also for maintaining homeostasis at the organismal level and its extensive involvement in human diseases. In mammals, FGF signalling possesses a large repertoire of 23 ligands and four canonical receptors. With the vast structural variations of heparan sulfates proteoglycans (HSPGs) as co-receptors, the combinatorial potential of the FGF signalling complex is enormous. On the other hand, FGF signalling is a member of the large receptor tyrosine kinase (RTK) signalling family, which is mediated primarily by Ras-MAPK, PI3 K-AKT and PLCγ-PKC pathways. Altogether, these features raise the question of how FGF signalling is deployed to meet its unique requirements in distinctive biological contexts.

**Figure 1.**
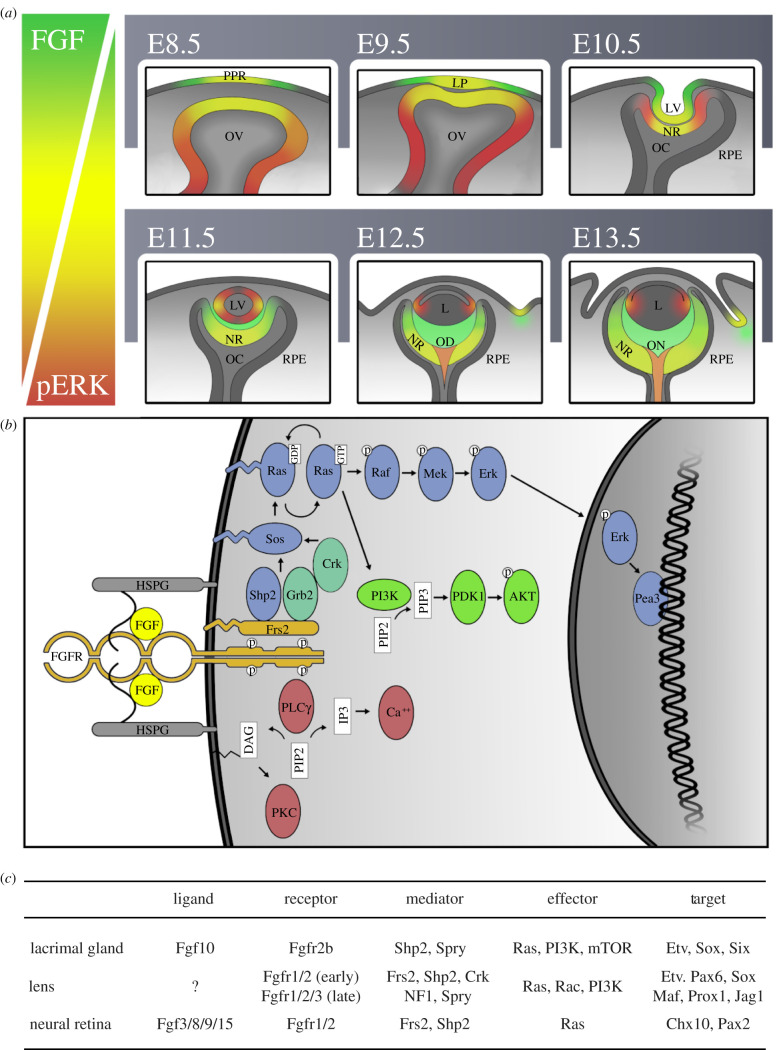
Temporal and spatial expression of FGF signalling during mouse eye development. (*a*) The dynamic expression of FGF ligands during the development of the mouse eye from E8.5 until 13.5 is depicted in green, while the expression of the FGF signalling downstream target pERK is shown in red, the overlap between the FGF ligands and pERK expression is highlighted in yellow. During early development at E8.5–E10.5, high levels of pERK and FGF secretion have been previously reported in the PPR, LP, OV, LV and NR. As development proceeds, pERK becomes restricted in the equator of the lens vesicle and the subsequent lens (L). In addition, pERK forms a high proximal to low distal gradient of expression in the retina of the optic cup that terminates at the junction of the RPE. Interestingly, at E12.5, high pERK activity has been reported in the OD and later the ON while little expression of FGF ligands in these regions has been reported. Finally, FGF signals from the mesenchyme stimulate the CE to develop into the LG. (*b*) Binding of FGF (yellow) to its receptor (beige) in the presence of HSPG (grey) causes the activation of three major downstream signalling cascades, the RAS-MAPK branch in blue, PI3 K-AKT in green and PLC*γ* in red. (*c*) Components of FGF signalling active in the LG, lens and NR, respectively.

The first visible sign of the eye during embryonic development is the optic sulcus, which is formed by bilateral evagination of the neural ectoderm at the eye field. As the optic sulcus expands laterally, the adjacent walls of the diencephalon in the optic field evaginate to become the optic vesicles (figure 1). This morphogenic event situates the optic vesicles in close proximity to the overlying ectoderm where the interaction between the neighbouring tissues induces development of the lens placode (LP), establishes the dorsoventral axis of the optic vesicle and regionalizes the neuroepithelium into the retinal pigmented epithelium (RPE), ciliary margin (CM), neural retina (NR) and optic disc (OD). Finally, the optic vesicle along the LP invaginates in coordination in order to generate the optic cup and the lens, establishing the blueprint for the developing eye.

In this review, we will focus on the eye as a model system to explore the functional diversity of FGF signalling. The eye is a complex organ that features many distinct yet highly conserved developmental niches; it is comprised the photosensitive posterior segment which is derived from the neural tube and the transparent anterior segment which originates from the surface ectoderm. The eye is also experimentally more accessible than internal organs or the brain, allowing for sophisticated genetic and pharmacological manipulations without jeopardizing animal survival. We will mainly focus on the role of FGF signalling in three unique components of the eye: the lacrimal gland (LG) as a one-dimensional model for budding morphogenesis to generate a glandular structure, the lens as a two-dimensional model for patterning and differentiation of a sensory placode, and lastly, the retina as a three-dimensional model for neurogenesis and homeostasis in the central nervous system. These three tissues showcase both the specificity and versatility of FGF signalling which enable it to play such important roles in biology.

## 2. The one-dimensional man: specific fibroblast growth factor signalling induces lacrimal gland budding

**Figure 2.**
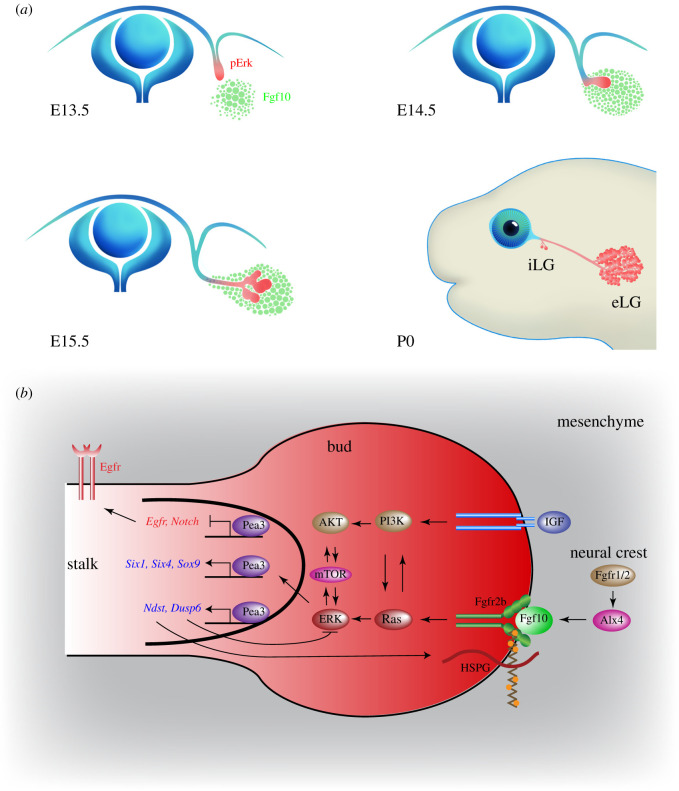
FGF signalling promotes the one-dimensional budding of the LG. (*a*) The LG originates from the thickening of the CE at E13.5, which becomes the LG bud at E14.5. Subsequent branching morphogenesis beginning at E15.5 generates both the intraorbital lacrimal gland (iLG) and extraorbital lacrimal gland (eLG) by P0. The Fgf10 gradient in the periocular mesenchyme is represented by green dots. The pERK activity in the LG epithelium is indicated in red. (*b*) The FGF signalling network in the LG bud. The neural crest FGF signalling induces Alx4 transcription factor to promote the expression of Fgf10 in the periocular mesenchyme, which binds to Fgfr2b in the presence of HSPG to induce Ras-ERK, PI3 K-AKT and mTOR in conjunction with IGF signalling. The Pea3 family transcription factors targeted by ERK activate Six1, Six4 and Sox9 to regulate LG differentiation, stimulate Ndst and Dusp6 to modulate the FGF feedback loop, and prevent aberrant Egfr and Notch signalling.

The LG is a tear-secreting organ which lubricates and protects the ocular surface. It begins to form on day 13.5 of mouse embryonic development (E13.5) with the thickening of the conjunctival epithelium (CE) at the temporal side of the eye [2] (figure 2*a*). This process is followed by the invasion of the epithelium into the surrounding mesenchyme at E14.5, with proliferating tip cells forming the ‘bud’ and differentiating follower cells forming the ‘stalk’. After E15.5, this elongated tubular structure undergoes the process of secondary branching morphogenesis, generating an increasingly complex LG. By postnatal day 0 (P0), the LG consists of the intraorbital and extraorbital lobes which are connected by a primary duct. Because the initial budding and elongation of the LG primordia occurs along a straight line, it serves as a one-dimensional model for studying the mechanisms of FGF signalling.

In the 1990s, Overbeek and his colleagues generated a series of transgenic mice to overexpress various growth factors in the lens, including EGF, FGF, IGF, PDGF, TGF and VEGF [3]. Among them, only *FGF3* induced intraocular glandular structures, complete with secretory acini and intralobular ducts [4]. This was later followed by FGF7 and FGF10, which, unlike FGF1, 2, 4, 8, 9 and 15, could force the corneal epithelium to adopt the glandular morphology [5,6]. As all three of these ligands belong to the FGF7 subfamily, it is clear that the gland-inducing capability is not coincidental. The definitive test came from analyses of mouse knockouts which showed that genetic deletion of *Fgf10*—but not *Fgf3* or *7*—abolished the LG [5,7]. Additionally, the essential role of *FGF10* in LG development has been confirmed by human genetics. Mutations disrupting FGF10 activity were found in patients suffering from aplasia of the lacrimal and salivary glands as well as lacrimo-auriculo-dento-digital (LADD) syndromes [8,9].

These findings beg the question: what makes FGF10/Fgf10 unique among growth factors to be both necessary and sufficient for the induction of development in the LG? Although we still don't have a complete answer to this fundamental question, recent efforts are beginning to reveal that the remarkable specificity of FGF10/Fgf10 signalling is an accumulative effect of ligand, receptor, cytoplasmic and transcriptional factors.

### 2.1. Ligand specificity

Timing and location are the first indications of Fgf10 signalling specificity in the LG. This is evident from the expression pattern of *Fgf10* mRNA, which first appears diffusively in the periocular mesenchyme at E12.5 and coalesces at E13.5 around the fornix of the CE where the future LG bud emerges [7,10] (figure 2*a*). Even as the LG bud begins to elongate away from the conjunctiva at E15.5, it still appears to be immersed in a cloud of *Fgf10*-expressing cells. By contrast, *Fgf7* never exhibits localized expression in the periocular mesenchyme [5]. These *Fgf10*-expressing cells are the descendants of neural crest cells which have migrated from the neural tube and are themselves regulated by FGF signalling [11] (figure 2*b*). The key player in mediating this relay of FGF signalling from the neural crest to the LG is the homeodomain transcription factor, Alx4, which when mutated not only abolishes the LG's *Fgf10* expression in mice, but also causes LG aplasia in humans. This Alx4–Fgf10 axis is probably active in other terrestrial animals that are exposed to dry air, as the Alx4-binding site in the *Fgf10* locus is present in animals such as chickens and lizards, but not in aquatic animals such as the *Xenopus* and zebrafish which do not require the tear-producing LG to moisten their eyes. This intriguing evolutionary conservation suggests that the Alx4–Fgf10 regulatory node may be an innovation of terrestrial animals as they moved from the water to the land.

The second advantage of Fgf10 for LG budding is its ability to generate a sharp morphogen gradient. Each member of the FGF ligand family is known to have different affinities to heparan sulfates, which are polysaccharide moieties of proteoglycans that are abundant in the extracellular matrix. This places variable restrictions on the diffusion of individual FGFs, which in turn form gradients at different steepness [12]. Fgf10 is a stronger binder to heparan sulfates than Fgf7 and, *in vitro*, produces a sharper gradient [13]. In explant cultures, this correlates to the propensity of Fgf10 to promote LG elongation and Fgf7 for branching. If, however, Fgf10 is mutated to weaken its binding to heparan sulfates, it can become an Fgf7-like molecule in both gradient profile and inductive effect. The same conversion can also result when degrading heparan sulfates in the LG culture, which further demonstrates the control of heparan sulfates on Fgf10 activity. In the most extreme case, Fgf10 becomes fully unmoored in the complete absence of heparan sulfates, making it impossible to maintain an effective local concentration sufficient for the induction of LG budding. This was observed *in vivo*, as the genetic ablation of heparan sulfates abolished LG development [10]. This phenotype was also reproduced by the inactivation of heparan sulfate *N*-deacetylase-*N*-sulfotransferase (Ndst) enzymes, which has a pleiotropic effect on the overall sulfation of heparan sulfates. By contrast, LG budding was unaffected by the loss of heparan sulfate 6-O and 2-O sulfation enzymes in the periocular mesenchyme, suggesting that the Fgf10 gradient is modulated by the overall negative charge of heparan sulfates rather than their sulfation sequences.

### 2.2. Receptor specificity

Among members of the FGF family, FGF10/Fgf10 is also distinguished by its receptor selectivity. In patients presenting with LADD syndrome, mutations in *FGF10* and *FGFR2* result in the same spectrum of abnormalities in the LG, ear, tooth and limb [9]. Similarly, *Fgfr2* conditional knockout mice phenocopy *Fgf10* null mutant mice with a LG aplasia defect [7,14]. These results suggest that FGF10/Fgf10 signals solely through the FGFR2/Fgfr2 pathway during LG development. The specificity of Fgf10 signalling is further enhanced by alternative splicing of *Fgfr2*, which toggles the third immunoglobulin (Ig)-like loop in the Fgfr2 ligand-binding domain between the ‘b’ and ‘c’ isoforms, making Fgfr2b the far more preferable receptor for Fgf10 [15]. Indeed, antisense oligonucleotides against Fgfr2b suppressed LG budding in explants [7]. Additionally, the alternative splicing of *Fgfr2* is developmentally regulated so that *Fgfr2b* and *Fgfr2c* are predominantly expressed in the epithelium and mesenchyme, respectively. If the mesenchyme expresses *Fgfr2b* instead of *Fgfr2c*, both *Fgf10* expression and LG development are abolished [16]. Thus, the tissue-specific distribution of Fgfr2 isoforms is critical for the targeting of Fgf10 signalling to the LG epithelium.

The next level of Fgf10 signalling attunement is implemented by HSPGs in the LG epithelium. Unlike their mesenchymal counterparts that interact with Fgf10 weakly to modulate its diffusion profile, the epithelial heparan sulfates act as co-receptors by forming a high-affinity complex with Fgf10 in the presence of Fgfr2b (figure 2*b*). This is necessary for triggering dimerization and auto-phosphorylation of Fgfr2b which initiates the intracellular signalling cascade. It thus places rather stringent requirements on the sulfation pattern of heparan sulfates, which are optimized for each Fgf/Fgfr pair. Although it remains debatable whether this selectivity constitutes a heparan code, heparan sulfate N, 6-O and 2-O sulfation enzymes have all been shown to be important for Fgf10/Fgfr2b signalling in the LG epithelium [14,17]. This is in contrast with what is described above in the mesenchyme, where heparan sulfate 6-O and 2-O sulfations are dispensable for LG development. Furthermore, N-sulfation of heparan sulfates is enriched in the bud of the LG, yet absent in the stalk, which correlates with the bud-specific activation of FGF signalling [14]. Conversely, inactivation of FGF signalling suppresses heparan sulfate N-sulfation, suggesting a positive feedback loop. Thus, heparan sulfate modifications contribute to the spatial confinement of FGF signalling to the LG bud.

### 2.3. Intracellular pathways

The FGF signalling gradient described above manifests in the LG epithelium as a graded pattern of Erk phosphorylation: high in the bud and low in the stalk [14]. Both Erk and its upstream kinase, Mek, are required to maintain bud cells in the undifferentiated and proliferative state in order to spearhead elongation [18,19]. This is mediated by Shp2, a non-receptor tyrosine phosphatase that plays a positive role in the Ras-MAPK pathway [20]. An important substrate of Shp2 in the LG bud is Spry2, which is induced by FGF signalling in a negative feedback loop and works to attenuate Ras signalling. The suppressor activity of Spry2 is normally reduced by Shp2 dephosphorylation. Thus, only a combined deletion of Spry2 and activation of Kras can compensate for the loss of Shp2 in LG induction. These results highlight the intricate cellular network that further fine tunes the Ras-MAPK activity in the LG bud.

The PI3 K-AKT pathway is another known target of FGF signalling. Biochemical studies have previously suggested that PI3 K is recruited to FGF receptors via the adaptor protein Gab1. However, the loss of *Gab1* does not affect either FGF signalling *in vitro* or LG induction *in vivo* [21]. Instead, the catalytic subunit of PI3 K contains a Ras-binding domain which mediates a direct interaction with Ras. Genetic abrogation of the Ras-PI3 K interaction completely abolishes FGF-induced AKT phosphorylation, suggesting that Ras is the bona fide mediator of FGF-PI3 K signalling (figure 2*b*). Although Ras plays a relatively minor role in the overall stimulation of PI3 K activity, the loss of Ras-PI3 K interaction still leads to patterning defects in the LG, as shown by an intrusion of EGF receptor expression from the stalk to the bud region. This is important because explant experiments have shown that over activation of EGF signalling interferes with LG budding. It further suggests that FGF-induced PI3 K signalling regulates the bud-stalk patterning of the LG.

### 2.4. Transcriptional effectors

The unique features of FGF10/Fgf10 signalling described above makes it ideally suited for the induction of branching morphogenesis, explaining why this pathway is deployed repeatedly in diverse organs such as the lung, pancreas and salivary glands. The prolific nature of FGF10/Fgf10 signalling suggests that it must also be flexible enough to function with local factors. The first category of such local agents is factors that establish the progenitor identity prior to the onset of Fgf10 signalling. Within the eye, both the LG and the harderian gland require Fgf10 signalling during development, but only the LG expresses Pax6. Mice carrying heterozygous mutations in the *Pax6* allele (*Sey^+/−^*) display severe LG deficiency, suggesting that *Pax6* may be the competence factor in the LG epithelium, shaping the response to Fgf10 signalling [7]. The second category of local agents is factors that are dependent upon FGF signalling. For example, FGF is required to maintain Sox9 expression in LG progenitor cells [22]. In return, Sox9 not only promotes FGF signalling by stimulating biosynthesis of heparan sulfates, but also cooperates with FGF signalling in order to induce expression of Sox10­, an essential regulator of acini differentiation. By acting as local interpreters, these transcription factors play crucial roles in orchestrating the LG specific response to FGF10/Fgf10 signalling.

How do these tissue-specific agents relate to FGF signalling? FGF signalling directly induces ETS domain transcription factors activated by MAPK phosphorylation [23]. Specifically, FGF signalling precedes the expression of the Pea3 (Etv) subfamily transcription factors, which are crucial for budding in the LG [18]. Transcriptomic analysis reveals the downregulation of many genes in *Pea3* mutants, including those involved in heparan sulfate modification and the regulation of MAPK signalling and even their own expression, reflecting the complex feedback interactions caused by FGF signalling (figure 2*b*). *Pea3* genes also regulate several transcription factors such as *Sox9*, *Six1* and *Six4*, all of which are targets of FGF signalling in the LG. By contrast, epidermal specific genes and Notch signalling were ectopically induced in the absence of *Pea3*, indicating aberrant progenitor fate determination*.* These results highlight the differential roles of the *Pea3* family genes in response to FGF signalling.

From the steep morphogen gradient to the concentrated downstream activity, the spatially directed Fgf10/Fgfr2b pathways promote the initial budding and continuous elongation of the LG. Fgf10/Fgfr2 also control the migration of eyelid tip cells by regulating the expression of Bmp, Shh, Activin and TGF*α* [24–26]. Fgfr2 plays important roles in the differentiation of the corneal epithelium and homeostasis of the Meibomian gland [27–29]. These results illustrate the versatility of FGF signalling, which we will elaborate in the following sections using the more complex systems of lens and retinal development.

## 3. The two-dimensional Janus: dynamic fibroblast growth factor signalling guides lens morphogenesis

**Figure 3.**
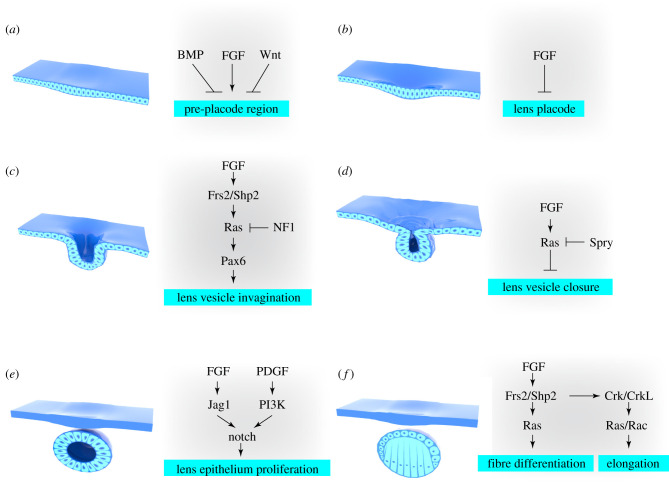
The timing and intensity of FGF signalling controls the two-dimensional patterning of the lens. The PPR is first selected from the head ectoderm by active FGF signalling devoid of suppressive BMP and Wnt (*a*), before progressing further towards the LP fate in lieu of continuous FGF signalling (*b*). FGF next induces Frs2–Shp2-mediated Ras signalling modulated by NF1 to promote Pax6 expression and lens vesicle invagination (*c*), but FGF signalling must be suppressed by Spry to allow lens vesicle closure (*d*). During the subsequent lens maturation, FGF cooperates with PDGF to stimulate Notch signalling, which promotes lens epithelium proliferation (*e*). In lens fibre cells, FGF signalling also activates Ras to promote differentiation and recruit Ras and Rac GTPases via Crk/CrkL to promote cell elongation (*f*).

Several sensory organs emerge during vertebrate development from cranial placodes, which are thickened epithelia within the lateral head ectoderm. In mice, the LP appears at E9.5 and forms the lens pit at E10.5, which develops into the lens vesicle (LV) at E11.5 after separation from the surface ectoderm. While the anterior LV cells maintain their epithelial form, the posterior cells differentiate and elongate into concentric rings of lens fibres packed with crystallin proteins (figure 3) [30]. Based on the rotational symmetry of the anterior–posterior axis, the lens can be depicted in a two-dimensional space. This relative structural simplicity presents an entry point to study the complexity of FGF signalling.

Research on cell signalling during lens development can be traced back to Hans Spemann's pioneering work with amphibians. After destroying the optic vesicle with a hot needle, Spemann showed that lens development was induced by a diffusible signal from the optic vesicle, a finding reminiscent of Spemann and Mangold's model of neural induction [31]. A century later, Hayashi *et al*. [32] found that a single injection of FGF2 was sufficient to induce an iris-derived second lens in an intact newt eye. Jane and Alfred Coulombre surgically rotated the chick lens 180 degrees, allowing for the posterior fibre cells to face the cornea, which ultimately resulted in an arrest of their growth [33]. By contrast, the anterior epithelial cells now exposed to the vitreous differentiated into new fibres. This elegant experiment demonstrated that the anterior–posterior polarity of the lens is also shaped by the ocular environment.

In this section, we will explore how repetitive use of FGF signalling guides consecutive phases of lens development. With a limited collection of communication channels, biological systems often reuse signalling pathways like FGF for diverse functions. The challenge is how to convey different message via the same signal. Just like the Roman god Janus presents two different faces in the front and the back, lens development elucidates how changes in the timing and intensity of FGF signalling can lead to distinct outcomes.

### 3.1. Lens induction

Beyond his findings on the tissue–tissue interactions underlying lens development, Spemann also proposed that the presumptive lens ectoderm must also acquire competency in order to respond to such inductive signals [34]. In chick embryos, both activation of FGF and the repression of BMP and Wnt are required to define the pre-placodal region (PPR) of the head ectoderm, which ultimately develops into the olfactory, auditory and LPs (figure 3*a*) [35]. While the LP is the default outcome of PPR definition, the olfactory placode emerges if FGF signalling is sustained [36] (figure 3*b*). FGF signalling must be turned on and then off for lens induction, which is further proven by lentoid derivation from human pluripotent stem cells [37,38].

Once the LP is specified, FGF signalling continues to play a crucial role in lens induction. Such can be seen in chick embryos, as the implantation of FGF8-containing beads has been shown to induce the lens marker *L-Maf* in the ectoderm [39]. Mouse genetics further identified Fgfr1 and 2 as redundant receptors for FGF signalling in this process, which is mediated intracellularly by the Frs2–Shp2–Ras-MAPK cascade (figure 3*c*). The disruption of Fgfr1 and 2, their co-receptors, HSPGs, or the interaction between Frs2 and Shp2 block normal lens induction [40–42]. Genetic ablation of Nf1, a major Ras GTPase, also prevents lens induction, which can be reversed pharmacologically by an MEK inhibitor administered *in vivo* [43]. This finding demonstrates that Ras activity must be precisely controlled to enable normal lens development. One important role of FGF signalling in lens induction is to prevent excessive cell death, since inhibition of the pathway results in extensive apoptosis [40,42]. However, the FGF pathway probably plays multiple roles in lens development. In chicks, the ectopic expression of *L-Maf* was induced by the application of FGF8, which suggests that FGF signalling regulates the expression of lens-specific transcription factors. The most critical target of FGF signalling is Pax6, a pivotal regulator of eye development [44]. Although FGF signalling does not affect the initial low level of Pax6 expression in the pre-placode phase, inactivation of FGF signalling prevents upregulation of Pax6 and its downstream targets, Sox2 and Six3 in the LP [40–42,45]. Furthermore, FGF is known to promote actin reorganization in lens cells through small Rho GTPases [46]. As actin-rich filopodia appear to tether the lens pit to the surrounding optic vesicle, the biomechanical role of FGF signalling in promoting the transition from the LP to the LV is worth investigating [47].

### 3.2. Polarization of the lens vesicle

After invagination of the lens pit, a transient structure known as the lens stalk connects the surface ectoderm to the LV. If the lens stalk remains, its presence may cause a lens-cornea attachment and result in a blurred cornea as shown in Peters anomaly, the human congenital disorder [48]. Much like the on-again/off-again pattern of FGF signalling during the LP development, research suggests that FGF signalling must be deactivated in the lens stalk to ensure the closure of the LV (figure 3*d*). This is shown by the presence of residual lens stalks after either overexpression of FGF in the retina or deletion of the FGF signalling inhibitors Spry1/2 in the lens [49,50]. The activation of Ras also induces this irregularity, which suggests that Ras pathway is probably the downstream effector [51]. The persistent lens stalk may be caused by lack of apoptosis, increased proliferation or altered cell adhesion in the junction between the surface ectoderm and LV. The dysregulating effect of hyperactive FGF-Ras signalling on these cellular processes requires further clarification.

The LV consists of anterior epithelial cells which proliferate and migrate towards the equator, as well as posterior epithelial cells which elongate towards the anterior rim and differentiate into primary lens fibres. Studies in rat lens explants have shown that FGF signalling has a dose-dependent effect on lens epithelial cells. At low concentrations, FGF promotes proliferation, while at high concentrations, it promotes differentiation [52–54]. These results point towards a model in which lens polarity is determined by an anterior^low^–posterior^high^ gradient of FGF signalling, which stimulates lens epithelial cells to proliferate in the anterior region and differentiate in the posterior region [55]. More recent studies have further revealed that lens patterning is refined by crosstalk between FGF, PDGF and Notch signalling (figure 3*e*). FGF signalling stimulates the posterior lens fibre cells to express Jag1, which in turn induces Notch signalling in the anterior lens to suppress the differentiation of lens epithelial cells [56,57]. Notch activity is enhanced intracellularly by PI3 K, a factor stimulated by PDGF signalling, and downregulated by FGF in the posterior lens, as the pathway suppresses the expression of PDGF receptors [58]. Disruption of FGF signalling by Shp2 deletion results in a posterior shift of the zone that divides the anterior and posterior lens, while blocking of the PDGF-PI3 K pathway causes an anterior shift. Thus, the balance of the FGF and PDGF pathways influences the anterior–posterior organization of the lens.

How does the anterior–posterior gradient of FGF signalling arise in the lens? As the Coulombre experiment suggests, the vitreous probably contains the FGFs that polarize the lens. However, their origin remains partly unclear. Transcripts of multiple FGF ligands have been detected in the lens, which may induce FGF signalling in an autocrine fashion [3]. On the other hand, FGFs may be released into the vitreous via the retina to promote lens development. This hypothesis is supported indirectly through the genetic deletion of *Lhx2* in the retina, which causes a reduction in retinal FGF expression and defective lens differentiation [50]. Various isoforms of FGF receptors are expressed in the spatially restricted pattern in the lens, and their co-receptors, heparan sulfates, also displayed in an anterior^low^–posterior^high^ gradient [59,60]. Together, they contribute to the overall pattern of FGF signalling—high in the posterior lens, low in the anterior lens and absent in the lens stalk. Nevertheless, among the 13 *Fgf* genes known to be expressed in the eye, none have been proven essential for lens development in mice [3]. This finding leaves the source of FGFs for lens patterning still unresolved.

### 3.3. Lens maturation

During the transformation from embryonic LV to adult lens, drastic changes in cellular content and size occur. This growth is enabled by the continuous proliferation of lens epithelial cells, which subsequently migrate to the posterior lens in order to differentiate into secondary lens fibres. At this final stage of development, the pattern of FGF signalling changes yet again, as Erk phosphorylation is weak in the anterior epithelium, strong in the transitional zone just above the lens equator, and reduced near the posterior and interior regions of the lens. Despite the weak activity in the anterior lens, FGF signalling is still required for the proliferation and survival of lens epithelial cells. The expression of dominant-negative FGF receptors or the conditional knockouts of critical FGF mediators, Frs2 and Shp2, led to increased cell death and reduced cell proliferation [20,45,61–63]. These outcomes are also observed after the deletion of *Fgfr2*, which demonstrates the importance of this receptor in the lens epithelium [59]. *In vitro* studies using lens explants indicate that both MAPK and PI3 K signalling mediate FGF-induced cell proliferation, which is especially sensitive to the duration of Erk phosphorylation [64,65]. This is consistent with previous findings that lens cell proliferation can be stimulated by the activation of Ras and reduced by the disruption of either MAPK or PI3 K [58,66–68]. MAPK and PI3 K also play important roles in the survival of the lens epithelial cells [58,68]. For example, Pten is generally known as a negative regulator of PI3 K signalling. However, its deletion can lead to increases in the activity of both MAPK and PI3 K in the lens as well as a significant rescue of the *Fgfr2* mutant phenotype [69,70]. Although FGF signalling preferentially stimulates Ras-MAPK over PI3 K-AKT, both pathways mediate FGF signalling in the lens epithelium.

The differentiation of lens epithelial cells commences in the transitional zone, which exhibits the strongest Erk phosphorylation. This process represents the combined efforts of three FGF receptors, as only the simultaneous deletion of *Fgfr1*, *2* and *3* can abrogate Erk phosphorylation and lens differentiation [71]. Similar to other optical regions, FGF-ERK signalling is supported by heparan sulfates, transmitted by the Frs2–Shp2 axis and modulated by inhibitors such as Spry and Sef [60,61,72,73] (figure 3*f*). In the transitional zone, however, FGF signalling promotes cell cycle exit by inducing the expression of cyclin-dependent kinases, such as p27 and p57, and lens differentiation genes, such as Prox1, c-Maf, Crystallins and aquaporin [59,74–77]. FGF signalling may also indirectly regulate lens differentiation gene networks through miRNAs [78]. The lens-specific role of FGF signalling may be largely attributed to cellular context, but it is also shaped by internal changes within the signalling cascade. For example, although the expression of the *Pea3* (*Etv)* gene family is under the control of FGF signalling, their deletion accelerates instead of prevents the differentiation of the lens epithelial cells, which indicates that these transcription factors have functionally diverged from the influence of FGF signalling in the lens [79]. The use of Crk family adaptors and stimulation of Ras and Rac1 activities by the core FGF signalling complex, Frs2–Shp2–Grb2, is another notable example of FGF reprogramming [80], which enables the elongation of fibre cells up to one thousand folds in length to constitute the mature lens. Finally, FGF controls and coordinates with other pathways such as Wnt, Yap, BMP and Notch during lens fibre differentiation [57,58,81–84]. Once the fibre cells are established, FGF-MAPK also promotes gap junction-mediated cell communication [85].

The development of the two-dimensional lens is considerably more complex than that of a LG bud and therefore requires a more dynamic cellular pattern. The on-again, off-again activity of FGF signalling from the pre-placode ectoderm, the LP, the LV to the mature lens demonstrates this complexity. The spatial pattern of the pathway becomes increasingly more complicated as the signalling intensity ranges from null in the lens stalk to low in the lens epithelium, and finally high in the transitional zone of the lens. Additionally, FGF signalling regulates other cellular processes such as proliferation, differentiation and cytoskeletal reorganization, as well as other signalling pathways. The multifaceted use of the FGF signalling pathway will be next illustrated through retinal development.

## 4. The three-dimensional virtuoso: diverse fibroblast growth factor signalling regulates retinal development and maintenance

**Figure 4.**
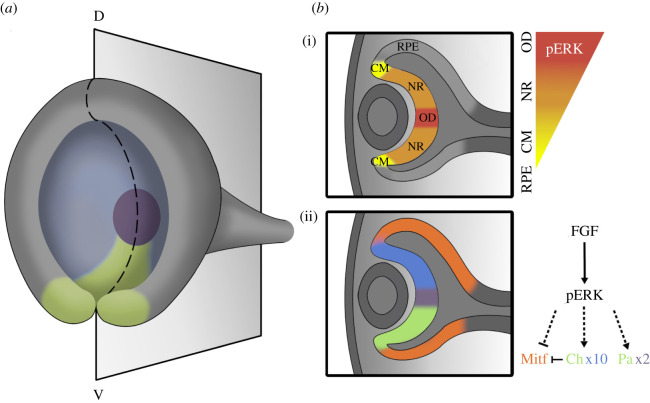
Functions of FGF signalling in the three-dimensional retinal development. (*a*) Schematic representation of the mouse E11.5 optic cup denotes the choroid fissure in green, the NR in blue and the OD in purple. D, dorsal; V, ventral. (*b*) (i) Cross-section of (*a*) illustrates the gradient activity of pERK in the establishment of the RPE, CM, NR and OD. LV, lens vesicle. (ii) FGF signalling promotes the neural retinal fate by activating the transcription factor Pax2 (green/purple) and Chx10 (green/blue) while also inhibiting the expression of the RPE specific factor Mitf (orange).

During the invagination of the optic vesicle, the first dorsoventral landmark emerges as a transient lateroventral groove in the optic cup, which is described as the choroid or optic fissure (figure 4*a*). Throughout development, this fissure expands medially while the ventral margins of the optic cup gradually surround it in a proximal-to-distal orientation. This event ultimately generates an opening at the OD for the entry of blood vessels and the exit of neuronal axons within the optic stalk. Based on its structure, the optic cup must be understood with a three-dimensional view.

Classic embryology shows the fluidity of optic cup development. In chick embryos, surgical removal of the distal cell layer of the eye cup, which typically gives rise to the NR, can lead to regeneration from the proximal RPE [86]. Transplantation of *Xenopus* optic cups with reversed orientation can transform the proximal RPE into a NR, whereas surgical removal of the surface ectoderm above the optic vesicle prevents NR differentiation [87,88]. When embryonic rat eyecups are cultured without the lens, the CM tissue is replaced with the NR [89,90]. These findings demonstrate that signals from the surface ectoderm and the lens induce the specification of the NR, CM and RPE.

In this section, we will present the diversity of FGF signalling as it structures and sustains the retina. FGF's influence not only affects developmental patterning and fate determination, but also homeostasis and metabolism.

### 4.1. Eye field specification

Eye field specification occurs prior to optic vesicle formation at the neural plate stage. This is initially evident through the activation of the eye field-specific transcription factors (EFTFs) including Rax, Pax6, Six3, Lhx2 and Six6. Studies in *Xenopus* have illustrated that inhibition of FGF signalling prior to the expression of Lhx2, reduced the population of the eye field cells [91]. However, this phenotype did not manifest when FGF signalling was inhibited at the late cleavage stages [91,92], suggesting a role of FGF signalling in the induction of the eye field but not its maintenance. By contrast, studies in *Xenopus* and zebrafish embryos have illustrated that overexpression of FGF reduces the expression of anterior neural tube markers and in turn the population of eye field cells [93,94]. However, it has previously been shown that overexpression of FGF resulted in delayed and abnormal epiboly during gastrulation in both *Xenopus* and zebrafish embryos. Therefore, the phenotypes resulting from the overexpression and inhibition of FGF during early development could be attributed to morphogenesis defects instead of lineage specification.

Following eye field specification, this tissue undergoes major morphological changes that give rise to the optic vesicles and subsequently the optic cups. Initially, a medial stream of cells, located posterior to the eye field, move anteriorly causing the central eye field to split into two bilateral retinal primordia. The bulk of previous research suggests that the separation of the single central eye field is mediated by Shh signals from the underlying mesoderm. Interestingly, inhibition of FGF signalling in *Medaka* fish and *Xenopus* embryos disrupt the separation of the eye field and cause cyclopia [95,96]. However, it remains unclear if cyclopia is caused directly by the lack of FGF in the migration of these cells or secondarily by the disruption of the diencephalic lineages and the dysregulation of Shh in embryonic mesoderm cells.

### 4.2. Axial patterning of the eye cup

Although the optic fissure is the first morphological landmark of the DV asymmetry of the optic cup, the establishment of this axis precedes its formation. This is evident through the uneven expression of transcription factors in the optic vesicle including Sox2, Pax2/6, Tbx5 and Vax1/2 which subdivide the NR into three main domains. *Fgf8* knockout zebrafish embryos (ace) exhibit a severe reduction in the size of the ventral retina [97]. Furthermore, a closer examination of *Xenopus* embryos treated with the FGFR inhibitor, SU5402, revealed a similar defect at the ventronasal portion of the retina and a concomitant expansion of the dorsal retina [98]. By contrast, over activation of FGF signalling in *Xenopus* embryos expanded the expression of the ventral retinal domain genes, *Vax1* and *Pax2,* dorsally. Similar to the DV axis, NT patterning occurs prior to the morphogenesis of the eye primordia, as evident by the distinct expression of *Foxg1* and *Efna5a*, which is restricted to the nasal domain, and *Foxd1* and *Epha4b* to the temporal region. Treatment of zebrafish embryos with SU5402 resulted in the expansion of *Epha4b* expression into the nasal region of the developing retina. Additionally, the combined deletion of *Fgf8*/*3*/*24* in zebrafish dramatically reduced the expression of both nasal specific genes, *Foxg1* and *Efna5a*, and expanded the expression of *Foxd1* and *Epha4b* [99,100]. Interestingly, a double deletion of *Fgf3* and *Fgf24* alone was not sufficient to disrupt normal NT axis formation. These results indicate that the *Fgf8* signal from the adjacent telencephalic vesicle is predominantly responsible for the patterning of the optic vesicle while the remaining Fgfs act redundantly to compensate for its loss.

Thus far, the only evidence in support of FGF signalling in axial patterning of the optic vesicle has come from zebrafish and frogs, which both differ significantly from mammals in the morphogenesis of the eye. Abnormalities in the establishment of a proper DV axis of the optic vesicle can cause coloboma, a common developmental defect where the failure of the choroid fissure fusion results in a gap at the ventral retina and consequently the iris. Studies using mouse genetics were the first to demonstrate that this phenotype can be reproduced when *Fgfr1* and *Fgfr2* are specifically deleted in the developing NR [101,102]. However, coloboma in these mutants resulted from the abnormal differentiation of the optic fissure margins which consequently disrupt its proper fusion [101]. Importantly, no axial polarity defects were observed. These findings suggest that the role of FGF signalling in the DV and NT patterning of the optic vesicle may not be conserved in mammals.

### 4.3. Regional specification of the optic cup

Since the initial eye cup transplant studies, it has been established that FGF is a major factor in the determination of the NR by inhibiting RPE differentiation (figure 4*b*). The expression of FGF occurs in the pre-lens ectoderm and the prospective NR after the contact of the optic vesicle to the surface ectoderm [103]. Indeed, the inductive properties of FGF in NR specification have been demonstrated in studies using explant cultures of optic vesicle [104,105]. Furthermore, ectopic expression of either FGF1, FGF2 or an activated allele of MEK1 using lentivirus infection and the removal of the surface ectoderm resulted in the depigmentation of the RPE [88,106,107]. The temporal requirement of FGF signalling was demonstrated in mice by genetic ablation of Shp2 using three retinal specific *Cre* drivers that were sequentially active at E8.5, E9.0 and E10.5. Although FGF-Ras signalling was eventually abolished in all three models, only the earliest deletion of Shp2 at E8.5 caused the presumptive NR cells to adopt the RPE fate [108]. FGF signalling has also been shown to stimulate the expression of Chx10 in the prospective NR which actively represses Mitf expression and inhibits the differentiation of the RPE lineage [108,109]. These experiments suggest that the delineation of the two optic cup lineages occurs at the optic vesicle stage in response to the FGF signals from the surface ectoderm.

If FGF signalling dictates the fate choice between NR versus RPE, how is the CM situated between these two regions specified? Our recent studies show that the retina expresses three FGF ligands, *Fgf3*, *Fgf9* and *Fgf15,* in a nested pattern, corresponding to a centre^high^–peripheral^low^ gradient of FGF signalling activity [110]. Flattening of the FGF gradient by sequential deletion of FGF ligands progressively impaired the subdivision of the CM, while the combined deletion of *Fgfr1* and *Fgfr2* completely abolished CM development, demonstrating that the FGF signalling gradient is required for the specification and subdivision of the CM. Moreover, FGF signalling regulates the activity of Wnt signalling, which is also required for CM development [111]. The synergy between FGF and Wnt signalling is revealed by a gain-of-function experiment, showing that constitutively active Wnt signalling transforms the NR into the CM or the RPE depending on the presence or the absence of FGF signalling, respectively [110]. This finding reveals a binary code of eye cup patterning in which the Wnt+/FGF− condition biases towards the RPE, Wnt+/FGF+ towards the CM and Wnt−/FGF+ towards the NR.

As previously mentioned, the junction of the optic stalk and the NR gives rise to the OD, a structure responsible for the proper axon guidance of the retinal ganglion cells (RGCs). Despite the proximity of the OD to the optic stalk, which is a source of *Fgf8* across most vertebrates [112,113], the OD exclusively generates astrocyte progenitor cells. The OD exhibits the strongest phospho-ERK activity in the entire optic cup. Fgfr1/2 or heparan sulfates knockout reduced the cell proliferation in the presumptive OD and ultimately caused their differentiation into the RPE [101,114]. Nevertheless, the overexpression of *Fgf8* failed to induce OD specific genes in the retina [113], suggesting that FGF signalling is necessary but not sufficient for OD development.

### 4.4. Neural differentiation

With the establishment of the optic vesicle cardinal axis, the neural progenitor cell population expands and differentiates into the six neuronal types that populate the retina. Much like the central nervous system, the NR initially develops as a pseudostratified neuroepithelium where the retinal progenitors undergo two waves of neurogenesis. The first wave of neurogenesis occurs in a centre-to-periphery manner where the majority of the differentiated RGCs first appear in the centre of the retina and gradually expand to the periphery as development proceeds [115]. At later stages of development, dividing cells in the inner nuclear layer migrate towards the outer layer as they begin to differentiate into the neuronal lineages. Notably, the differentiation of the neuronal lineages that populate the retina unfolds in a sequential manner. In particular, the first type of neurons to emerge is the ganglion cells followed by the amacrine cells, cone photoreceptors and the horizontal cells. The bipolar cells and rod photoreceptors emerge postnatally.

There is ample evidence that FGF signalling controls the proliferation of retinal progenitor cells (PRCs). This was first observed in rat retinal explants, in which PRCs showed increased proliferation when subjected to FGF [116]. Furthermore, *in vivo* studies using *Xenopus* embryos illustrated that the inhibition of FGFR1 dramatically decreased the production of neuronal cells [117]. Cell cycle genes were also drastically reduced in the murine retina after genetic deletion of either *Fgfr1* and *Fgfr2* or *Frs2* and *Shp2* in the retina, demonstrating that PRC proliferation is controlled by the FGF–Frs2–Shp2 pathway [101,102,108]. Recent single-cell RNAseq analysis has provided further insight into the dynamics of PRC regulation by FGF signalling [110]. Multiple groups of PRCs identified in the S phase eventually entered the G2/M phase and gave rise to either retinal neurons or CM cells. Interestingly, trajectory analysis has shown that neurogenic progenitors in the G2/M phase irreversibly progress towards terminal differentiation, whereas the CM progenitors in the G2/M phase can either differentiate or return to the S phase. Inactivation of FGF signalling not only reduced the population of PRC clusters, but also biased the CM progenitors to exit the cell cycle. This suggests that FGF signalling promotes the self-renewal of PRCs and prevents their immature differentiation.

The more controversial question is whether FGF signalling directly controls differentiation of retinal neurons. A number of studies in chicks and zebrafish have provided evidence for FGF involvement in RGC differentiation. In particular, treatment of the optic vesicle explants with FGF8 or FGF1 has been shown to promote RGC differentiation, while exposure of the explants to SU5402 dramatically inhibited the expression of proneural genes, slowed down the centre-to-periphery differentiation wave and reduced the number of RGCs [113,115,118]. Treatment of RGC cultures with FGFs has been shown to stimulate axonal sprouting and growth, while inhibition of this signalling pathway caused defects in their axon extension [119,120]. Finally, at later stages of development, studies in chicks have revealed a focal expression of *Fgf8* in the so-called rod-free zone (RFZ), which shares intriguing characteristics with the fovea region in primates. This RFZ was lost after siRNA mediated knockdown of *Fgf8*, suggesting that FGF signalling may prevent rod photoreceptor differentiation in this region [121]. A confounding factor in the interpretation of these results is the overlapping role of FGF signalling in PRCs and in neuronal differentiation as any perturbation of mother cells may indirectly affect their progenies. As mentioned above, we have addressed this question by generating the temporal deletion of Shp2 using distinct Cre drivers that act sequentially during retinal development [108]. Our results show that only the earliest deletion of Shp2 at the OV stage disrupted the establishment of the RPC fate, which led to the loss of retinal neurons. Despite the high efficiency of Cre activity and clear reduction of pERK, no retina neural differentiation defects were observed in the two late onset Shp2-deletion models. In line with these findings, our recent single-cell analysis shows that the loss of FGF signalling disrupted PRC proliferation and CM differentiation, but the neurogenic progenitors or differentiated neurons were unaffected. These results suggest that FGF signalling may function differently between mammals and lower organisms during retinal neurogenesis.

### 4.5. Retinal vascularization

As one of the most metabolically active tissues in the body, the retina is nourished by two independent vascular networks, the retinal vessels and the choroid. Within the ocular system, FGF2 stimulates angiogenesis in the choroid, as over-expressions of FGF2 in the retina cause a latent proangiogenic phenotype in mice [122,123]. On the other hand, transgenic mice that overexpressed a dominant-negative FGFR1 in the RPE suffered from poorly branched vascular bed in the choroid, absence of retinal primary vascular plexus and failure of hyaloid vessel regression [124,125].

It was initially reported that the deletion of *Fgfr1* and *2* in endothelial cells did not affect developmental angiogenesis or basal vascular permeability in the retina [126]. Instead, neovascularization induced by laser-induced choroidal injury or oxygen-induced retinopathy was impaired, suggesting that FGF signalling may be a therapeutic target for pathological angiogenesis. More recent studies showed that the endothelial inactivation of *Fgfr1* induced the expression of *Fgfr3* and the combined deletion of *Fgfr1* and 3 resulted in significant impairment of retinal angiogenesis [127]. This defect is caused by the reduced expression of the glycolytic enzyme HK2, which led to the downregulation of glycolytic flux and glucose uptake. These findings reveal metabolism as another target of FGF signalling in regulating cell proliferation and migration.

### 4.6. Neuroprotection

While the majority of the studies focus on the diverse role of FGFs during the development of the eye, there has been increasing evidence of this signalling pathway in regulating ocular homeostasis. In particular, studies in zebrafish have illustrated that the blocking of FGF signalling in the adult retina results in the degeneration of photoreceptors [128]. However, such phenotypes have not been observed in mammals either due to a compensation from other neurotrophic signals in higher vertebrates or due to fundamental differences in neural tissue homeostasis. Nevertheless, there is increasing evidence to suggest a potential role of FGFs in the neuroprotection of the eye. For instance, RPE cultures are shown to secrete Fgf2 in response to chemically induced oxidative stress [129]. In addition, optic nerve (ON) injury in rats causes the upregulation of Fgf2 mRNA in photoreceptors as well as an upregulation in diabetic retinopathy in rats [130,131]. Finally, a concomitant upregulation of Fgfr1 expression is observed in photoreceptors following focal injury as well as induced retinal detachment [132].

Due to the increase in Fgf2 secretion in response to injury, it is strongly suggested that a similar neuroprotective role of FGFs may occur in the adult retina. Indeed, studies using retina explants derived from rats with retinal dystrophy displayed a dramatically decreased degeneration of photoreceptors when exposed to FGF2 and CNTF [133]. In addition, *in vivo* studies also demonstrated the neuroprotective potential of FGF signalling in response to light-induced injury as the exogenous supply of FGF2 could reduce the photoreceptor depletion [134]. Interestingly, this effect of FGF2 has also been observed in the survival of RGC following ON section suggesting a pan-neuronal influence [135]. It should be considered that the neuroprotective role of FGFs is not restricted to FGF2, as exogenous FGF1 and FGF21 were also shown to reduce the degeneration of photoreceptors [136,137]. While the previously mentioned studies provide strong evidence of FGF neuroprotection, it remains unclear if this is a direct effect as the underlying mechanism of this effect has yet to be elucidated.

### 4.7. Retinal regeneration

FGFs's ability to regenerate after retinal injury can be seen in studies with zebrafish and chicks. After light-induced injury in zebrafish, retinal regeneration was enhanced by intravitreal FGF2 injection [138]. Interestingly, FGF2 injection in the absence of injury fails to induce the proliferation of Müller cells, which suggests that the pathway is activated with other cytokines in response to injury [138]. Indeed, interleukin-6 and FGF2 demonstrated a synergistic role in inducing Müller cell proliferation in uninjured retinas of teleost fish [139]. Yet, the effect of FGF stimulation appears to be age-dependent in fish, as forced *Fgf8a* expression promotes the proliferation of Müller cells in youth, but inhibits their proliferation in old age [140]. Regardless, retinal regeneration has been shown to be dramatically reduced when FGF signalling is inhibited [128]. However, this finding is in conflict with similar studies which reveal only a moderate reduction of Müller cell regeneration [138]. The variability of these experimental results points to the partial nature of FGF inhibition, which may cause distinct phenotypes. Much like zebrafish findings, chicken animal models also illustrate increased stimulation of Müller cell regeneration in response to excitotoxic damage following FGF2 injections [141]. Cell proliferation appears to be mediated by the MAPK downstream branch of FGF signalling [142]. The proliferative potential of FGF is enhanced when combined with retinoic acid activation, which further suggests FGF alone is insufficient for retinal regeneration and probably acts in combination with other signals to initiate this process [141].

While the previously mentioned studies provide strong evidence for the regenerative potential of FGF, both animal models have the capacity of spontaneous regeneration. The capacity of FGF to activate Müller cell regeneration is lost in mammals. No regeneration was recorded upon the exogenous supply of FGFs [134]. Additionally, injections of FGF2 alone following excitotoxic injury did not promote regeneration in rat retinas [143]. Nevertheless, FGF2 has the potential to accelerate the proliferation of Müller cells derived from rat retinas *in vitro* [144]. Moreover, *in vivo* studies demonstrate that FGF2 could stimulate retinal regeneration in combination with insulin or Wnt signalling activator CHIR99021 [145,146]. Therefore, it is possible that other signals could prime the Müller cells to acquire stem cell characteristics where FGFs could act as mitogens in order to drive their proliferation.

## 5. Conclusion

The difficulty of believing that a perfect and complex eye could be formed by natural selection, though insuperable by our imagination, should not be considered subversive of the theory. Charles Darwin*, The Origin of Species*

Darwin found the eye to be the most challenging structure to explain in his theory of evolution. Today, the molecular understanding of eye development and homeostasis remains far from complete. In this review, we have presented three vignettes to showcase how a single intercellular communication pathway like FGF signalling can make such diverse, multifaceted contributions to ocular development. In the one-dimensional model of LG budding, FGF signalling achieves remarkable specificity based on selective ligands, receptors, intercellular mediators and transcriptional targets. In the two-dimensional model of lens morphogenesis, FGF signalling dynamically manipulates timing and intensity in order to generate distinctive outcomes. In the three-dimensional model of the retina, FGF signalling demonstrates its versatility in regulating patterning, neurogenesis, neuroprotection and regeneration. Many important questions still remain to be answered. For example, we still lack a complete understanding of how FGF signalling is transduced intracellularly, including the interaction between Fgf receptors to their downstream adaptors, the interactome of key mediators such as Shp2, and the ultimate effectors that produce transcriptional and cytoskeletal changes in the eye. Although *in vitro* studies have indicated that FGF signalling can be modulated in both magnitude and frequency, the current model of FGF function in the eye remains largely static. Lastly, despite the rich milieu of intercellular signals *in vivo*, our understanding of the crosstalk between FGF and other signalling pathways is still rather rudimentary. Nevertheless, we have come a long way since the time of Darwin in establishing the molecular foundation for eye development and function. Further studies of FGF signalling will undoubtedly enrich our understanding of the eye as a paradigm of organ development and maintenance.

## Data accessibility

This article has no additional data.
